# Field-scale detection of Bacterial Leaf Blight in rice based on UAV multispectral imaging and deep learning frameworks

**DOI:** 10.1371/journal.pone.0314535

**Published:** 2025-01-17

**Authors:** Guntaga Logavitool, Teerayut Horanont, Aakash Thapa, Kritchayan Intarat, Kanok-on Wuttiwong

**Affiliations:** 1 School of Information, Computer and Communication Technology, Sirindhorn International Institute of Technology, Thammasat University, Pathum Thani, Thailand; 2 Advanced Geospatial Technology Research Unit, Sirindhorn International Institute of Technology, Thammasat University, Pathum Thani, Thailand; 3 Department of Geography, Faculty of Liberal Arts, Thammasat University, Pathum Thani, Thailand; 4 Reserach Unit in Geospatial Applications (Capybara Geo Lab), Faculty of Liberal Arts, Thammasat University, Pathum Thani, Thailand; 5 Rice Department, Bangkok, Thailand; Shandong Agricultural University, CHINA

## Abstract

Bacterial Leaf Blight (BLB) usually attacks rice in the flowering stage and can cause yield losses of up to 50% in severely infected fields. The resulting yield losses severely impact farmers, necessitating compensation from the regulatory authorities. This study introduces a new pipeline specifically designed for detecting BLB in rice fields using unmanned aerial vehicle (UAV) imagery. Employing the U-Net architecture with a ResNet-101 backbone, we explore three band combinations—multispectral, multispectral+NDVI, and multispectral+NDRE—to achieve superior segmentation accuracy. Due to the lack of suitable UAV-based datasets for rice disease, we generate our own dataset through disease inoculation techniques in experimental paddy fields. The dataset is increased using data augmentation and patch extraction methods to improve training robustness. Our findings demonstrate that the U-Net model incorporating ResNet-101 backbone trained with multispectral+NDVI data significantly outperforms other band combinations, achieving high accuracy metrics, including mean Intersection over Union (mIoU) of up to 97.20%, mean accuracy of up to 99.42%, mean F1-score of up to 98.56%, mean Precision of 97.97%, and mean Recall of 99.16%. Additionally, this approach efficiently segments healthy rice from other classes, minimizing misclassification and improving disease severity assessment. Therefore, the experiment concludes that the accurate mapping of the disease extent and severity level in the field is reliable to accurately allocating the compensation. The developed methodology has the potential for broader application in diagnosing other rice diseases, such as Blast, Bacterial Panicle Blight, and Sheath Blight, and could significantly enhance agricultural management through accurate damage mapping and yield loss estimation.

## Introduction

Thailand plays a critical role in the global rice market, consistently ranking among the top ten producers and exporters from 2018 to 2023 [1]. Nevertheless, Thailand faces significant challenges, ranking in the highest quartile of countries vulnerable to hazards such as droughts, floods, and epidemics [2]. Among these, one of the most concerning is epidemics, particularly crop diseases. Bacterial Leaf Blight (BLB) is among three major severe rice diseases [3] caused by the bacterium *Xanthomonas oryzae* pv. *oryzae* (Xoo), which spreads through several vectors, including wind-driven precipitation, irrigation water, and direct contact between plants [4]. The symptoms appear as long lesions on the leaf edges, which can extend to cover the entire leaf area, leading to tissue death [5]. BLB usually attacks rice in the flowering stage and can cause yield losses of up to 50% in severely infected fields [6, 7]. The resulting yield losses severely impact farmers, necessitating compensation from the regulatory authorities. Accurate detection of BLB is crucial to ensure that compensation to farmers is properly allocated based on the precise extent of the disease in each field. Therefore, this study aims to develop an automated detection system for BLB in a rice field using aerial imagery and deep learning (DL) frameworks.

The advancement of remote sensing technology has enabled the use of unmanned aerial vehicles (UAVs) in smart farming and precision agriculture. UAV offers flexible flight adjustments, very-high spatial resolution, and prompt data acquisition without the need for revisits like those required in satellite systems [8]. These capabilities make UAVs particularly valuable for tasks such as monitoring growth status [9], assessing crop biomass [10], and predicting yields [11]. UAVs can also be equipped with various sensors, showcasing advanced approaches in crop monitoring, especially for optimizing disease detection [12–14]. For example, Albetis et al. [15] utilized UAVs with multispectral sensors for crop disease analysis, demonstrating their effectiveness in detecting plant diseases and pests. Research by Zhang et al. [16] further supported the efficiency of multispectral sensors in identifying various plant health issues. Additionally, Mirandilla et al. [17] discovered that sensors operating in specific spectral bands, such as red, red-edge, and near-infrared, are capable of detecting and assessing the severity of major rice diseases, including BLB, blast, and tungro.

Vegetation Index (VI) techniques are instrumental in analyzing vegetation’s physiological status, enabling precise assessments of crop health, growth, and stress [18, 19]. Among these, the Normalized Difference Vegetation Index (NDVI) [20] and the Normalized Difference Red Edge Index (NDRE) [21] are well-known VIs commonly used in remote sensing vegetation analysis. In recent years, these indices have been applied to rice disease image recognition due to their simplicity, effectiveness, and strong correlation with disease severity. For instance, Zhang et al. [22] found that image-based NDVI measurements correlate well with the severity of sheath blight disease. Similarly, Ainunnisa and Haerani [23] demonstrated that NDRE is effective in identifying rice diseases and pests, such as rice blasts, due to its ability to detect chlorophyll levels in rice plants. In practical applications, VIs provide a more comprehensive evaluation than relying solely on the raw spectral system for detecting crop pests and diseases [24].

In recent years, DL-based Convolutional Neural Networks (CNNs) have been widely used in remote sensing imagery to extract various spatial features. CNNs require less feature engineering than traditional methods, which are often more complex and heavily dependent on the user’s expertise. Additionally, traditional methods frequently require manual adjustments when the dataset changes, highlighting their lack of adaptability compared to more automated approaches like CNNs. As a result, employing CNNs can simplify complex processes and enhance performance outcomes [25]. The layers within CNNs can be configured in various architectures, each optimized for specific tasks.

U-Net is a pixel-based semantic segmentation architecture that involves classifying each pixel of an image into distinct categories based on their coherence or contrast with other regions. U-Net fundamentally comprises two main parts: (i) the contracting path (encoder), responsible for feature extraction, and (ii) the expansive path (decoder), which synthesizes predictions [26]. These two network segments are arranged in a U-shaped configuration, symbolizing the architecture’s name. The encoder part of U-Net is highly customizable and can be configured with different backbone architectures to enhance performance on various tasks. For crop disease feature extraction, Zhang et al. [27] employed a U-Net baseline model for the semantic segmentation of wheat yellow rust disease in UAV multispectral images. Their findings indicated that the U-Net model was effective in detecting and classifying the severity of wheat yellow rust at field scales, achieving an overall accuracy of 96.93%. Similarly, Oliveira et al. [28] compared two state-of-the-art semantic segmentation architectures, PSPNet and U-Net, for detecting nematode disease in coffee crops in Brazil. Their study found that U-Net provided superior performance, with a 73% accuracy based on the F-measure metric. Su et al. [29] further applied a U-Net architecture in an automated rust disease monitoring framework using multispectral UAV images, achieving a high accuracy of 94.8% in detecting and classifying rust severity levels in wheat. Recent studies collectively demonstrate the efficacy of U-Net in crop disease detection, particularly in its ability to accurately segment disease-affected areas. However, the baseline U-Net architecture exhibits certain limitations when applied to more complex and high-resolution remote sensing data. Specifically, U-Net may struggle with capturing fine-grained details and distinguishing between subtle variations in vegetation, which are critical for precise BLB detection. The relatively shallow nature of its encoder limits the model’s capacity to extract hierarchical and abstract features, especially in scenarios where BLB manifestations are nuanced or vary significantly across different rice fields or environmental conditions [30].

Recent research has increasingly turned to enhanced U-Net variants that incorporate deeper and more powerful backbones, such as Residual Neural Network (ResNet) [31, 32] to address the limitations of the baseline U-Net architecture. ResNet is a popular choice for customizing the U-Net encoder, introduced by He et al. [33]. This architecture leverages residual blocks with skip connections, effectively mitigating the issue of vanishing gradients during deep network training. These skip connections are strategically implemented at intervals of two or three layers, each configured with ReLU activation and batch normalization layers. Typically, ResNet begins with a single 7x7 convolution kernel, followed by max pooling with a 3x3 kernel and a stride of 2, and concludes with average pooling. The framework is adaptable and is offered in several variants such as ResNet-18, ResNet-34, and ResNet-101, differentiated by the number of residual blocks. When configured as the backbone of U-Net, ResNet enhances the model’s ability to extract and refine features, leading to improved segmentation performance in complex tasks. The use of a ResNet-101 backbone within the U-Net framework allows for more robust feature extraction by leveraging the residual connections and depth of ResNet. This not only improves the model’s ability to capture complex patterns and multi-scale features but also enhances its overall segmentation accuracy [34].

Despite the advancements in UAV and DL-based approaches for various crop diseases, there is a notable absence of research focusing on UAV and DL applications for BLB disease detection. Specifically, there are no existing studies that implement automated UAV and DL pipelines for detecting BLB, particularly in regions like Thailand. Moreover, while vegetation indices (VIs) such as NDVI and NDRE have been widely used in crop health monitoring, their integration into UAV-based DL frameworks for BLB detection remains unexplored. This lack of research presents a significant gap in the current literature, highlighting the need for dedicated studies to explore the potential of UAV, VIs and DL approaches in BLB detection and management. Therefore, our study implements UAV and DL approaches on a multispectral dataset combined with VIs to address this gap and enhance BLB detection capabilities.

The main contributions of this study are as follows:

1. We implement a U-Net architecture with a ResNet-101 backbone in UAV-based multispectral imagery for BLB detection. The multispectral imagery in our study include RGB bands along with red edge (RE) and near-infrared (NIR) bands.
2. We evaluate the performance of the multispectral image and two other combinations: multispectral + NDVI, and multispectral + NDRE, to determine which setup yields the best results.
3. This study offers an efficient strategy using knowledge-based systems for agricultural management and loss assessment systems.

## Materials and methods

### Overview

**Fig 1:**
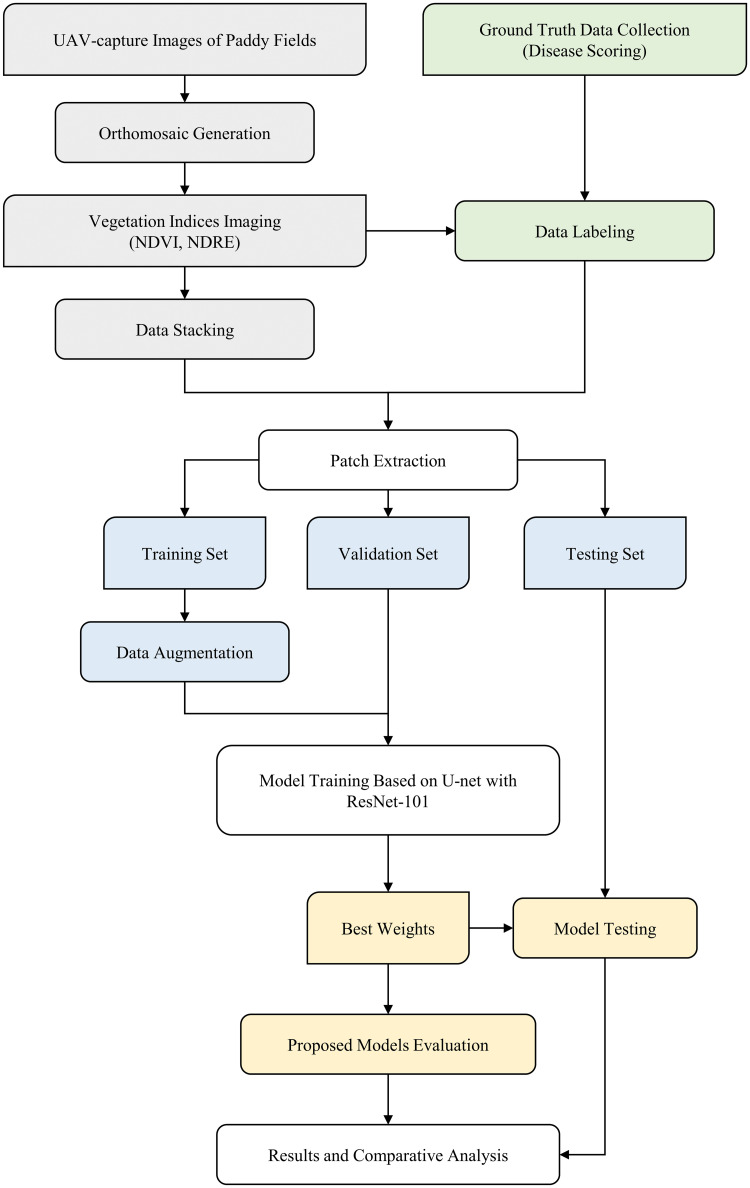
Workflow of field-scale detection of BLB in Rice based on UAV multispectral imaging and DL frameworks.

Fig 1 illustrates the research plan of our experiment. Initially, UAV-captured images of the paddy field are stitched together to create an orthomosaic. In parallel, vegetation indices (VIs) such as NDVI and NDRE are computed, and the resulting data are stacked to build different dataset combinations. These datasets, along with their corresponding labels, are divided into patches (available at https://doi.org/10.6084/m9.figshare.26955862.v1), which are subsequently split into training, validation, and test sets. Data augmentation is applied to the training set to enhance the robustness of the model. The three sets are then used to train a U-Net model with a ResNet-101 backbone across different band combinations. The model is first trained and validated, and the best-performing model weights are selected for final testing. The results from the proposed models are evaluated and compared to determine the most effective approach for classifying the severity levels of BLB-infected rice fields.

### Study areas

**Fig 2:**
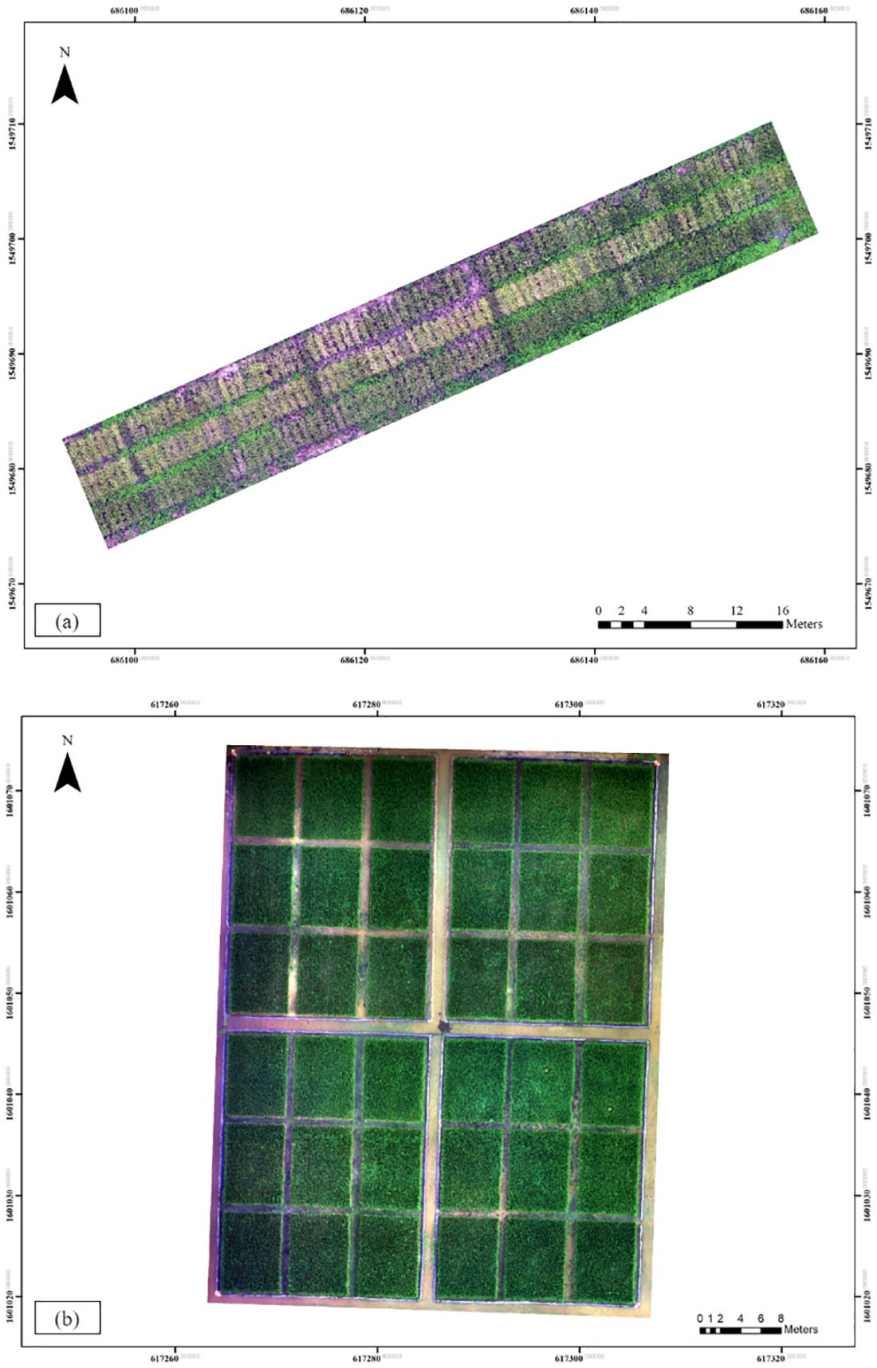
Mapping of the study areas. (a) Pathum Thani Rice Research Center, and (b) Thailand Rice Science Institute.

The experiment is conducted in two study areas, illustrated in Fig 2. Firstly, the Pathum Thani Rice Research Center is referred to as study area A (14.0169° N, 100.7295° E). Secondly, the Thailand Rice Science Institute is designated as study area B (14.4763° N, 100.0893° E). Both study areas are located in the central region of Thailand, where rice cultivation is the predominant agricultural activity, accounting for more than 50% of the region’s annual economic crop production.

**Fig 3:**
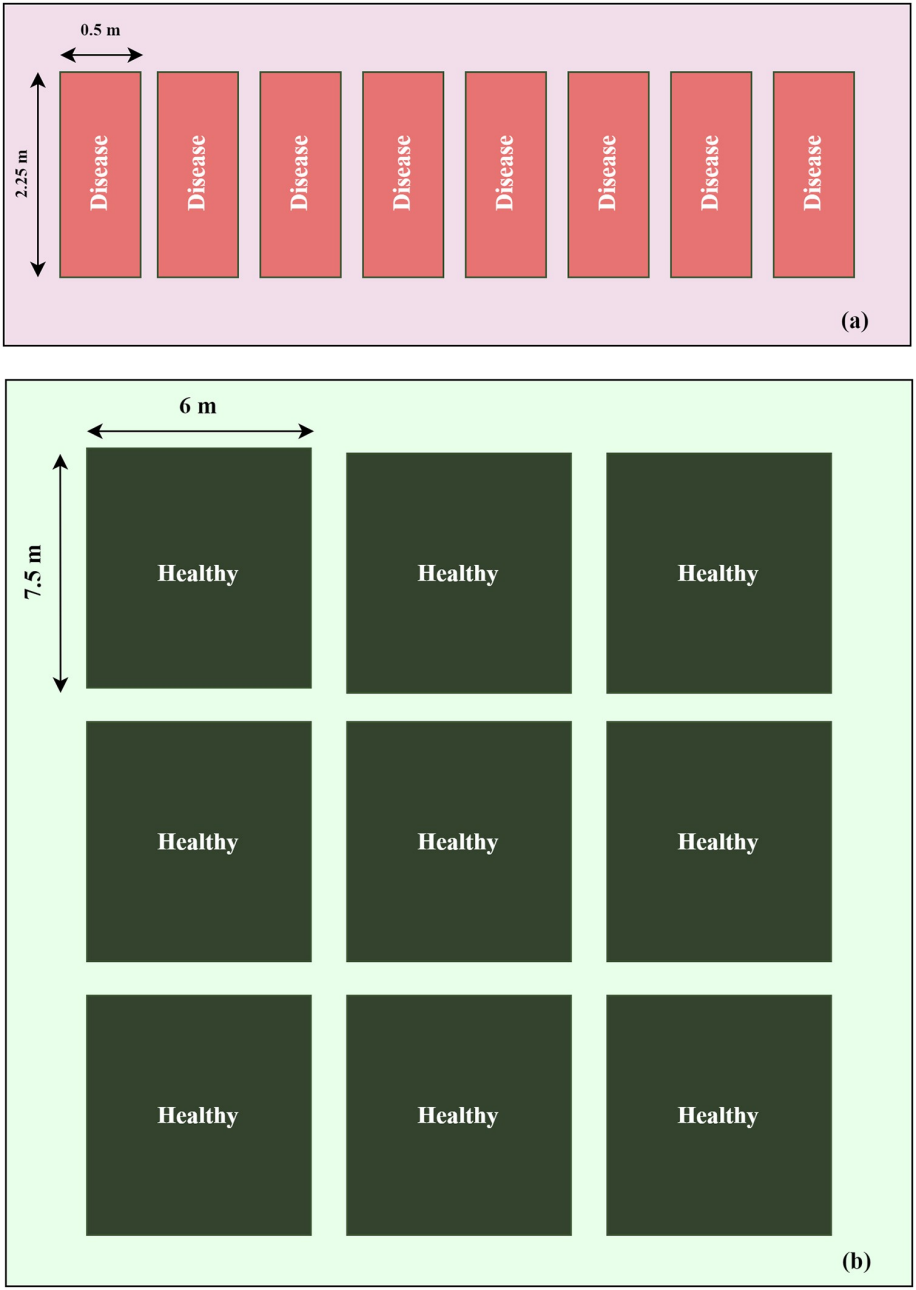
Example layout of experimental subplots in the paddy field. (a) Study area A, and (b) Study area B.

In study area A, the rice plants are organized into 234 subplots distributed across three baselines. Each subplot measures 0.5 × 2.25 m (1.15 m²). This area is specifically designated for collecting data on rice affected by BLB. Additionally, we implement a disease inoculation method based on clipping. This technique involves dipping scissor tips into *Xoo* and cutting the leaf tips during the booting growth stage. Meanwhile, the paddy rice field in study area B consists of 36 subplots, each measuring 6 × 7.5 m (45 m²). This area is used to collect data on healthy rice. The layouts for both study areas are illustrated in Fig 3. All other management procedures adhere to the protocols established by the Rice Department of Thailand.

### UAV imagery and ground-truth data collection

**Fig 4:**
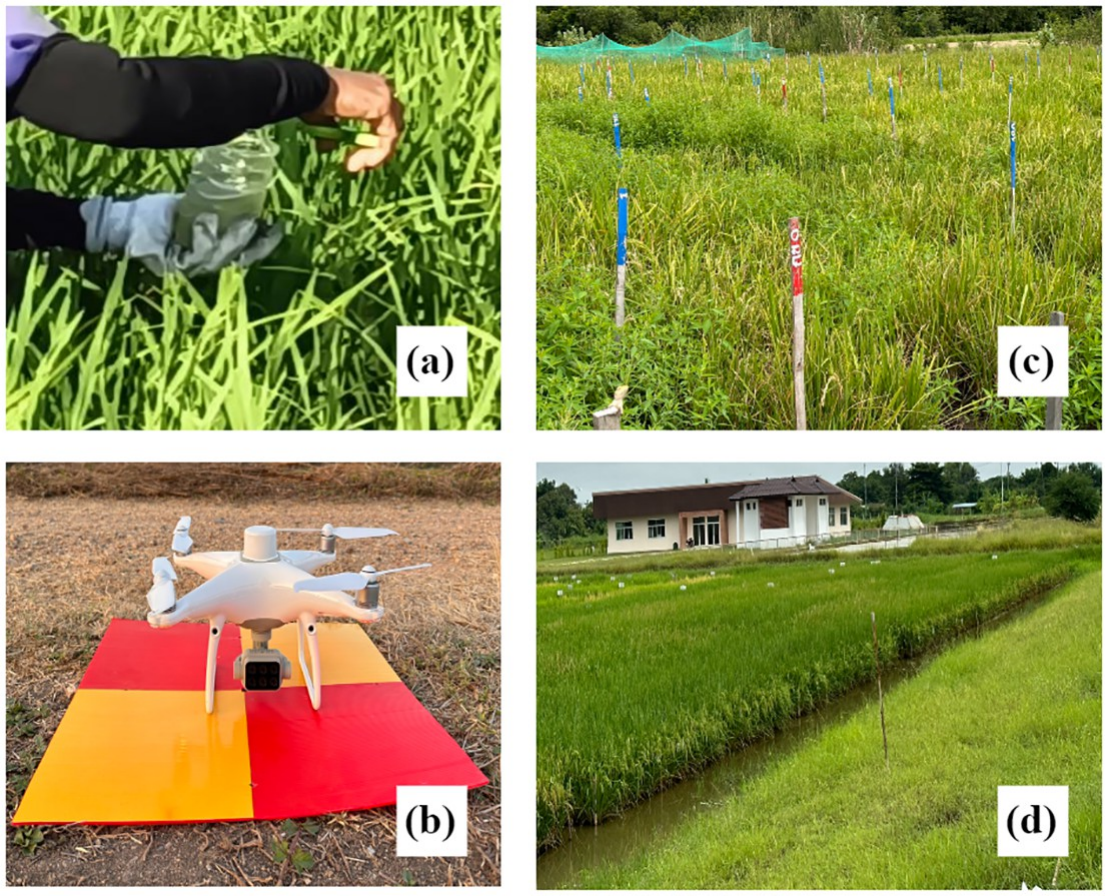
Field surveying photos in our experiment. (a) Disease Inoculation, (b) DJI P4 Multispectral, (c) Study area A, and (d) Study area B.

In this study, the raw dataset for both healthy and diseased paddy fields is collected using the DJI Phantom 4 (P4) multispectral drone, manufactured by Shenzhen DJI Sciences and Technologies Ltd. This quadrotor UAV is equipped with six 1/2.9-inch CMOS sensors covering specific spectral bands: blue (450 ± 16 nm), green (560 ± 16 nm), red (650 ± 16 nm), red-edge (730 ± 16 nm), near-infrared (840 ± 26 nm), and visible light (RGB). A height of 20 meters, widely used in DL methods for UAV-based crop disease detection [35], is set for image capture. The flight parameters are detailed in Table 1. Five ground control points (GCPs) are established with Real-time Kinematic (RTK) for geo-referencing, using the World Geodetic System (WGS) 1984 datum and Universal Transverse Mercator (UTM) Zone 47 North projection. Furthermore, the surveying photos during our experiment are shown in Fig 4.

**Table 1:** Flight parameters for UAV data collection.

Parameter	Value
Camera Model	P4 Multispectral Camera
Shooting Angle	Course Aligned
Capture Mode	Hover Capture at Point
Flight Course Mode	Inside Mode
Time of Flights	11:00 a.m. (clear sky)
Speed	15 m/s
Height	20 meters
Resolution	1.1 cm/px
Front Overlap Ratio	70%
Side Overlap Ratio	70%
Course Angle	110°

Ground-truth measurements, particularly disease scoring based on the IRRI Standard Evaluation System for rice [5], are applied by rice disease experts to estimate the extent of damage and severity of BLB disease. This assessment is conducted 21 days after disease inoculation, using a scale of 1, 3, 5, 7, and 9, each corresponding to percentages ranging from 1% to 100%, as shown in Table 2.

**Table 2:** BLB disease scoring for ground truth data collection.

Score	Extent of leaf area affected by BLB
1	BLB affecting 1% to 5% of the leaf area
3	BLB affecting 6% to 12% of the leaf area
5	BLB affecting 13% to 25% of the leaf area
7	BLB affecting 26% to 50% of the leaf area
9	BLB affecting more than 50% of the leaf area

### Orthomosaic generation

The related scene images from each raw dataset, are used to create image models, including geo-referencing, dense cloud, mesh, and digital elevation models (DEM), using Pix4Dcapture version 2.0.18 software. Pix4Dcapture facilitates the stitching of individual images into a comprehensive orthomosaic using these models. The orthomosaic images, comprising blue, green, red, red-edge, and NIR bands, are resized within the focused area boundaries and normalized to a range of 0-1 using the min-max scaling method to support model training. These outputs are then utilized for further analysis and information extraction in subsequent processing steps.

### Vegetation indices imaging

In our experiment, we compute two vegetation indices—the NDVI and the NDRE—to enhance the performance of BLB disease extraction. NDVI indicates the presence and density of healthy green vegetation. NDRE, on the other hand, is particularly sensitive to chlorophyll concentration and is more suited for identifying plant stress earlier. Both indices have been widely used in agricultural monitoring to assess crop health and detect early signs of disease [23, 36]. NDVI and NDRE can be expressed as follows:

$$\begin{array}{ccc} \text{NDVI} & = & \frac{\text{NIR}-\text{R}}{\text{NIR}+\text{R}} \end{array}$$ (1)

$$\begin{array}{ccc} \text{NDRE} & = & \frac{\text{NIR}-\text{RE}}{\text{NIR}+\text{RE}} \end{array}$$ (2)

where *NIR*, *R*, and *RE* represent near-infrared, red, and red-edge bands, respectively.

### Data stacking and labeling

**Fig 5:**
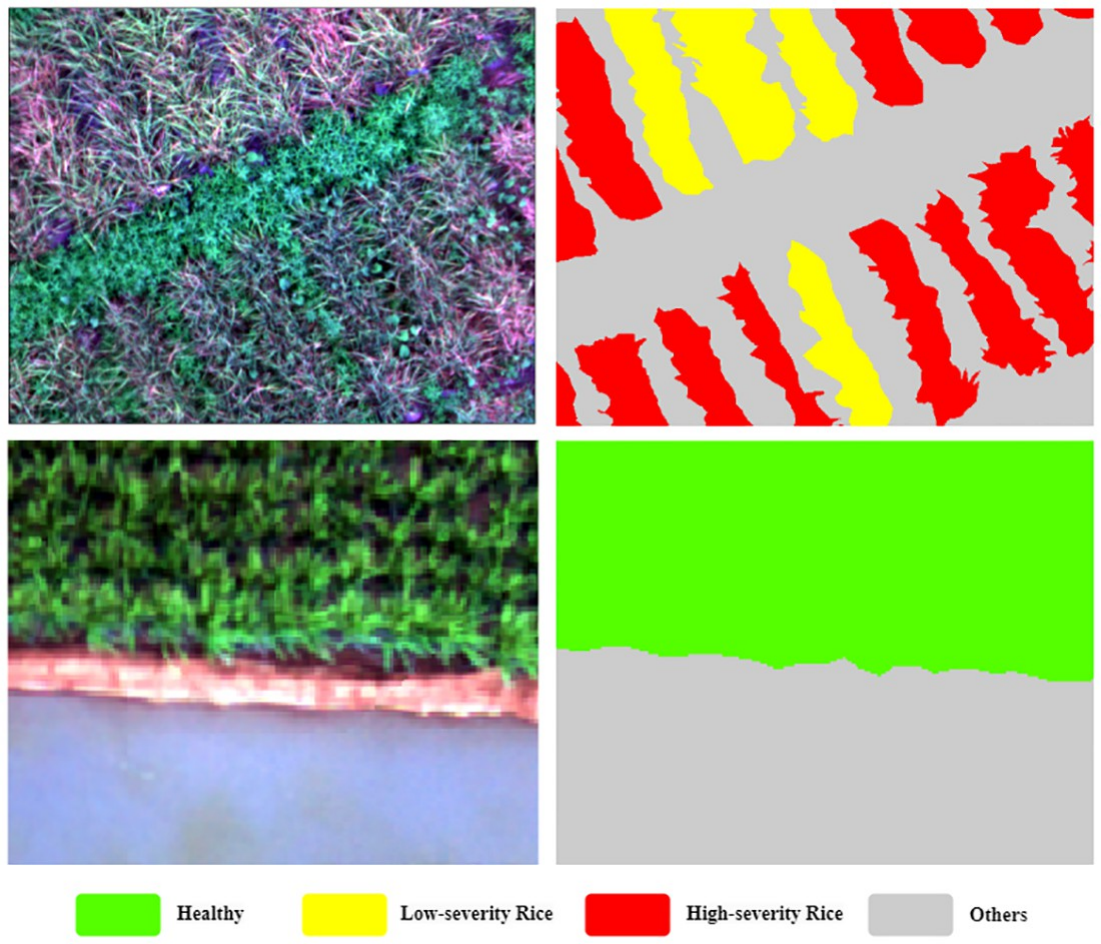
Sample image and corresponding label used for training the proposed models.

After VIs computing, we investigate three combinations of image band stacking: multispectral (D1), multispectral with NDVI (D2), and multispectral with NDRE (D3). Regarding the classification, we propose four label classes: healthy rice, low-severity BLB (subplot affected 1–50%), high-severity BLB (subplot affected more than 50%), and other non-rice categories such as soil, weeds, and water, as illustrated in Fig 5.

### Patch processing

Each image dataset, along with its corresponding labels, is divided into patches of 256 × 256 pixels using an integrated overlap function of 128 × 128 pixels, strategically employed to significantly increase the dataset size, which is crucial for effective model training. Zero-padding is applied to ensure the dimensions are divisible as required. After extracting the patches, the dataset is separated into training, validation, and testing sets with a ratio of 70:15:15, typically comprising 1,299 images for training, 277 for validation, and 281 for testing. An augmentation technique with a 75% probability is applied exclusively to the training dataset to ensure robustness in model training.

### Model training based-on U-Net with ResNet-101

**Fig 6:**
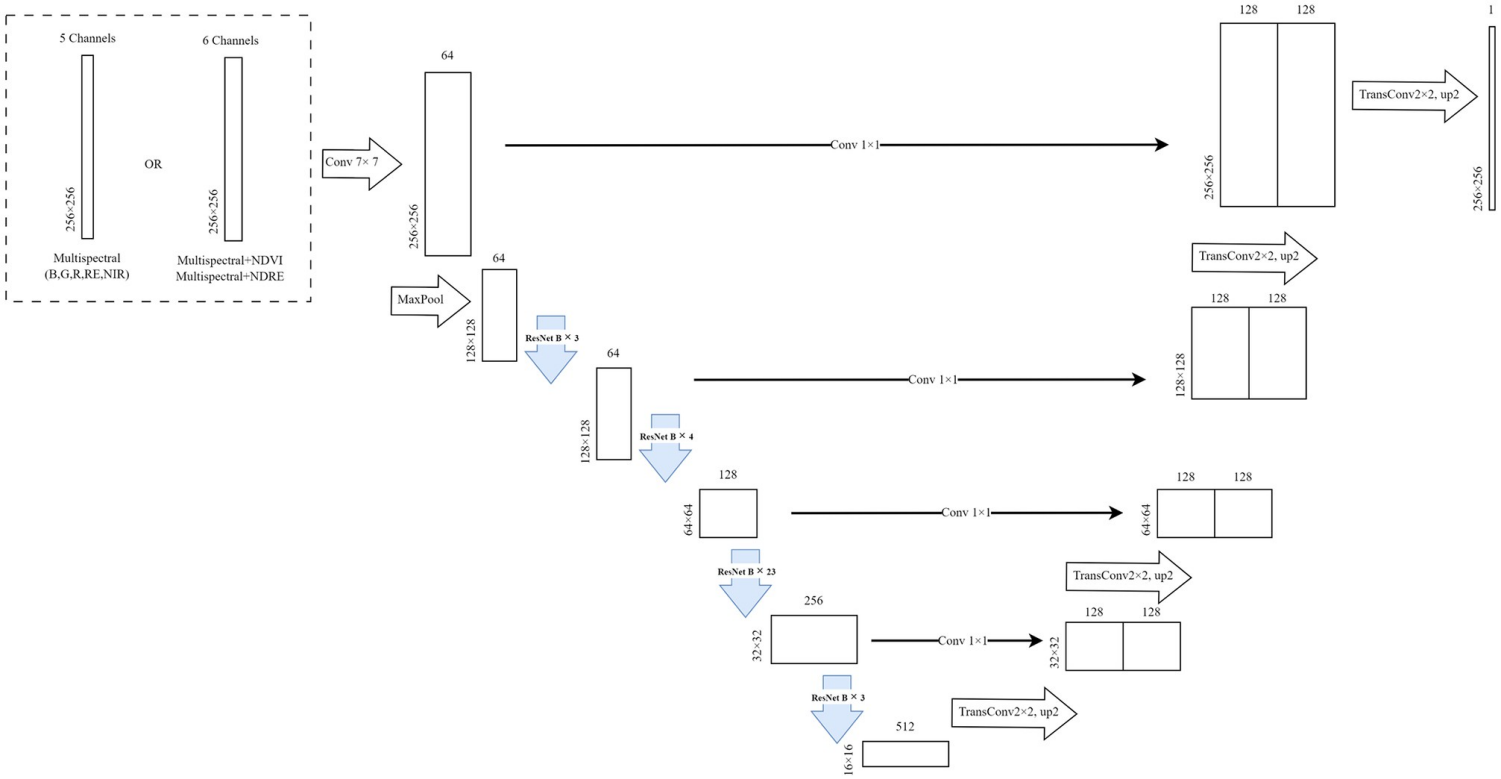
Framework of the proposed U-NET with ResNet-101 backbone.

In this study, we develop an automated model for detecting BLB in UAV imagery by employing U-Net with ResNet-101. ResNet-101, pre-trained with ImageNet weights, serves as the backbone to enhance the feature extraction capabilities of the model. A 7 × 7 multispectral convolution layer is implemented at the forefront of the architecture to effectively handle the multi-band dataset. This initial layer is crucial for capturing both spatial and spectral details necessary for accurate disease detection. The framework of the proposed models is illustrated in Fig 6.

Cross-entropy loss is applied to optimize the models for the proposed datasets (D1-D3). Cross-entropy loss is commonly used in DL for classification tasks and has proven effective in pixel-wise classification for semantic segmentation. It is particularly useful for multi-class problems, as it minimizes the difference between predicted and actual class probabilities, making it suitable for the relatively balanced portions of our dataset [37]. The Adam optimizer, with a learning rate of 0.001, is used to train the models. Additional training parameters are configured as detailed in Table 3. The following parameters are used to optimize each model to its best weights based on the highest Intersection over Union (IoU) values obtained during validation.

**Table 3:** Training parameters for model training.

Parameter	Value
Class	4
Encoder	ResNet-101
Encoder weight	ImageNet
Activation function	Sigmoid
Batch size	16
Input patch size	256 × 256
Epoch	500
Optimizer	Adam
Learning rate	0.001
Loss function	Cross-entropy

The training is conducted on a system equipped with an Intel Xeon E5-2696 v2 CPU @ 2.50 GHz, an NVIDIA GeForce GTX 1080 Ti GPU, 64 GB of RAM, running Windows 10 Pro, and using PyTorch version 2.0.0.

### Model evaluation and performance analysis

We evaluate and compare the performance of our proposed models based on different band combinations employing several key accuracy metrics, including Intersection over Union (IoU), Accuracy, and F1-score. The equations for these metrics are provided below:

$$\begin{array}{c} \text{IoU}=\frac{\text{TP}}{\text{TP}+\text{FP}+\text{FN}} \end{array}$$ (3)

$$\begin{array}{c} \text{Accuracy}=\frac{\text{TP}+\text{TN}}{\text{TP}+\text{TN}+\text{FP}+\text{FN}} \end{array}$$ (4)

$$\begin{array}{c} \text{F}1-\text{score}=2\times \frac{\text{Precision}\times \text{Recall}}{\text{Precision}+\text{Recall}} \end{array}$$ (5)

where

$$\begin{array}{c} \text{Precision}=\frac{\text{TP}}{\text{TP}+\text{FP}}\;\text{and}\;\text{Recall}=\frac{\text{TP}}{\text{TP}+\text{FN}} \end{array}$$ (6)

True positive (TP) occurs when both the actual and predicted classes are positive, while true negative (TN) occurs when both the actual and predicted classes are negative. False positive (FP) occurs when the actual class is negative but the predicted class is positive. Similarly, false negative (FN) occurs when the actual class is positive, but the predicted class is negative.

## Results and discussion

### Results

#### Statistical results and visualization of the proposed method

U-Net models, each equipped with a ResNet-101 backbone, are trained using the same training parameters listed in Table 3. These models are then evaluated to determine the best-performing model based on the highest Intersection over Union (IoU) score achieved during validation. This evaluation process results in three proposed models. Specifically, models M1 to M3 represent U-Net configurations with ResNet-101, utilizing different combinations of input datasets: multispectral (D1), multispectral with NDVI (D2), and multispectral with NDRE (D3), respectively. The training for each of the models (M1, M2 and M3) take an average of 9.27 hours.

**Fig 7:**
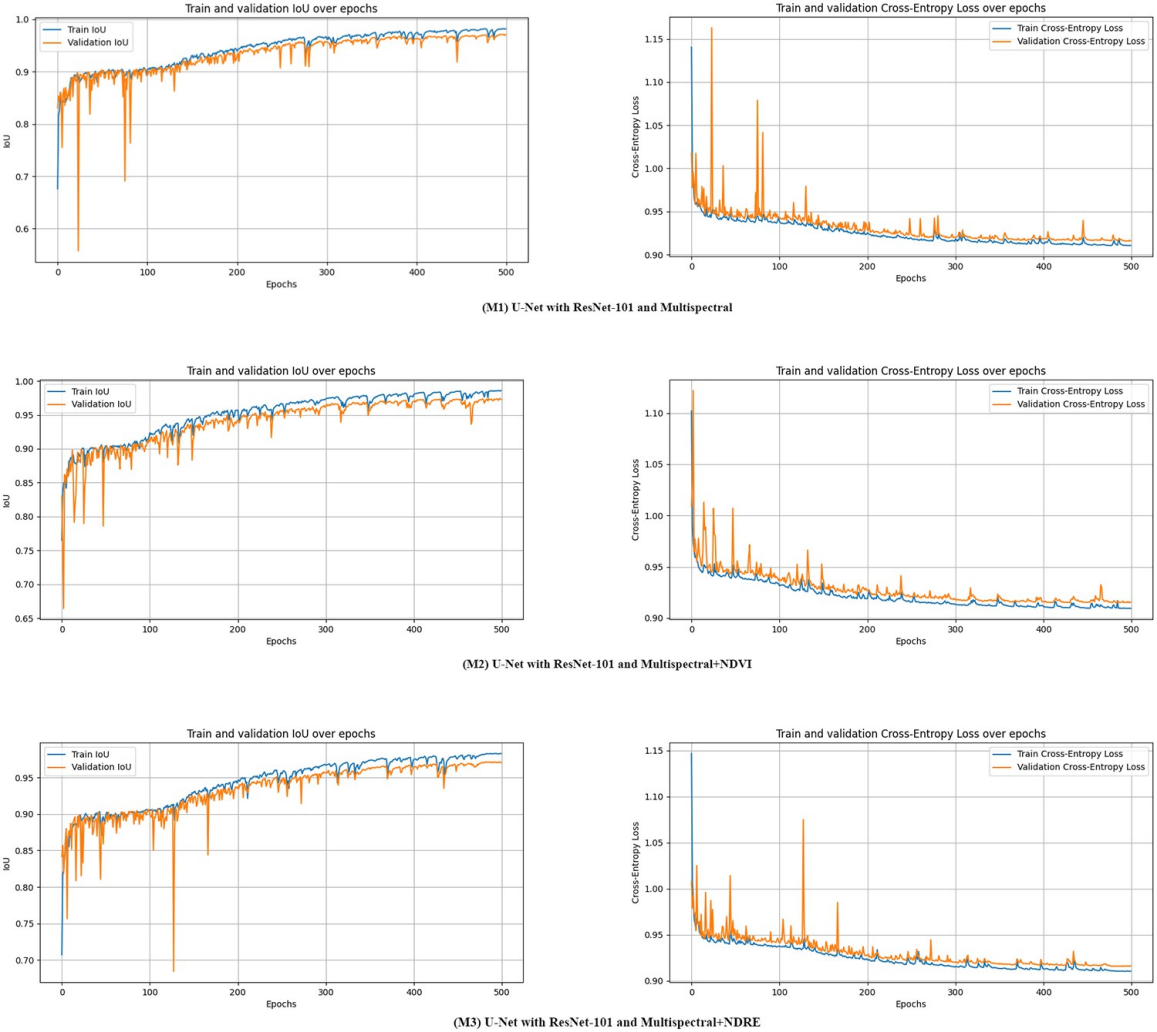
Performance curves metrics over epochs for proposed models.

Fig 7 presents the performance curves for U-Net models with ResNet-101 using different input datasets: D1 (M1), D2 (M2), and D3 (M3) over training and validation epochs. Initially, the IoU scores for the training sets of M1, M2, and M3 are 67.63%, 76.51%, and 70.74%, respectively, while the IoU scores for the validation sets are 83.11%, 83.01%, and 84.21%, respectively. When the models achieved their best IoU performance, the IoU scores for the training sets of M1, M2, and M3 are 98.22%, 98.58%, and 98.29%, respectively, and the IoU scores for the validation sets reach 97.14%, 97.40%, and 97.13%, respectively. These results demonstrate that M2 achieves the highest IoU score during both training and validation, outperforming the other proposed models.

After achieving their best weights, our proposed models are subsequently evaluated on unseen test sets, with the results presented in Table 4. These experiments demonstrate high performance in detecting BLB in UAV images, achieving mean IoU (mIoU) scores ranging from 96% to 99%, mean F1-score (mF1) from 98% to 99%, and mean accuracy (mAccuracy) from 99% to 100%. Additionally, the models exhibit strong precision and recall, with mean Precision (mPrecision) ranging from 97% to 98% and mean Recall (mRecall) from 99% to 100%. The average inference time across all three models is 1.21 seconds per iteration (s/it) for each image.

**Table 4:** Performance of U-Net with ResNet-101 backbone using different combinations of input datasets. Note that multispectral, multispectral with NDVI, and multispectral with NDRE, models referred to as M1, M2, M3, respectively.

Model	mIoU (%)	mAccuracy (%)	mF1 (%)	mPrecision (%)	mRecall (%)	Inference Time (s/it)
M1	97.05	99.39	98.48	97.81	99.17	1.08
M2	97.20	99.42	98.56	97.97	99.16	1.04
M3	96.98	99.37	98.44	97.98	99.11	1.51

M2 outperforms the other proposed models across all accuracy metrics, achieving 97.20% mIoU, 99.39% mAccuracy, an mF1 of 98.56%, an mPrecision of 97.97%, and an mRecall of 99.16%, with an inference time of 1.04 seconds per iteration (s/it). Meanwhile, M3 has the slowest inference time at 1.51 s/it. However, the least effective model, M3, still performs commendably, achieving 96.98% mIoU, 99.37% mAccuracy, an mF1 of 98.44%, an mPrecision of 97.98%, and an mRecall of 99.11%. In addition, M1 closely matches the results of M3, with 96.98% mIoU, 99.37% mAccuracy, an mF1 of 98.44%, an mPrecision of 97.81%, and an mRecall of 99.17%.

**Fig 8:**
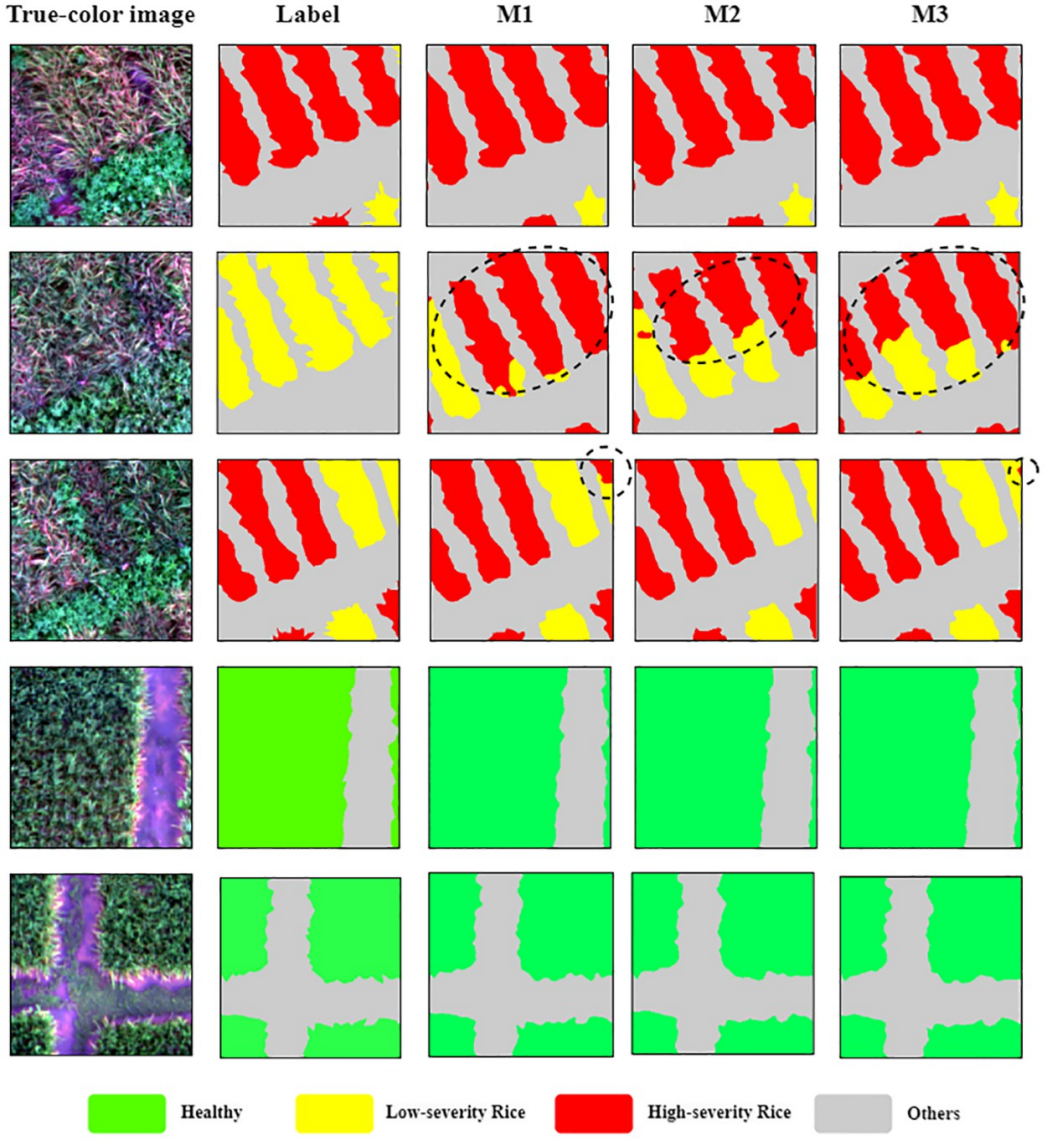
Comparison of visual classification results with test UAV datasets for BLB disease in rice detection.

Fig 8 illustrates the visual classification results of the three proposed models when apply to example test sets, display alongside true-color composites (RGB) and ground-truth labels for comparison. The different colors in the label images signify the classified pixels of each category: green represents healthy rice, yellow represents low-severity BLB in rice, red represents high-severity BLB in rice, and gray denoted other classes such as soil, weeds, and water. The first two rows mainly displays high-severity and low-severity BLB in rice plots. The third row primarily showcases all diseased and other classes, while the fourth and last rows involve healthy classes. As observed in examples from the test sets, M2 most accurately identified the severity levels, avoiding mixed pixels within classes and minimizing misclassification. Additionally, this model effectively segments rice in individual plots from complex non-vegetative categories, specifically weeds. In contrast, the other models (M1 and M3) occasionally misclassify between low-severity and high-severity classes and struggle with the connectivity of each boundary in rice plots compared to the ground-truth images. This indicates that M1 and M3 offer less precision in assessing and detecting severity in rice, despite showing high accuracy metrics. However, all the proposed models effectively segment the healthy rice class.

#### Comparative analysis with other methods

Table 5 illustrates the performance comparison between U-Net with ResNet-101 backbone (ours), U-Net with MobileNetV2 [38] backbone, and DeepLabV3+ [31] with ResNet-101 backbone across three different dataset combinations (D1-D3). Our proposed model outperforms both models for all datasets. It consistently demonstrates superior performance. Additionally, the U-Net with MobileNetV2 backbone performs better than DeepLabV3+ with the ResNet-101 backbone, highlighting that U-Net offers a more effective architecture than DeepLabV3+. However, the ResNet-101 backbone provides better feature extraction and robustness than MobileNetV2, making it a preferred choice for our U-Net model.

**Table 5:** Comparison of our proposed model with other models in three different combinations of datasets.

Dataset	Architecture	Backbone	mIoU (%)	mAcc (%)	mF1 (%)	mPrecision (%)	mRecall (%)	Inf (it/s)
D1	U-Net	ResNet-101	97.05	99.39	98.48	97.81	99.17	1.08
D2	U-Net	ResNet-101	97.20	99.42	98.56	97.97	99.16	1.04
D3	U-Net	ResNet-101	96.98	99.37	98.44	97.98	99.11	1.51
D1	U-Net	MobileNetV2	96.02	99.16	97.94	96.89	99.03	1.25
D2	U-Net	MobileNetV2	96.20	99.20	98.40	97.02	99.08	1.22
D3	U-Net	MobileNetV2	96.11	99.18	97.99	96.97	99.04	1.36
D1	DeepLabV3+	ResNet-101	95.04	98.94	97.40	96.34	98.50	1.26
D2	DeepLabV3+	ResNet-101	95.53	99.05	97.67	96.71	98.65	1.08
D3	DeepLabV3+	ResNet-101	95.18	98.97	97.48	96.43	98.56	1.69

*Note:* D1, D2, and D3 refer to different combinations of spectral bands: D1 is Multispectral, D2 is Multispectral combined with NDVI, and D3 is Multispectral combined with NDRE. The term “mAcc” denotes mean accuracy, and “Inf” denotes inference time.

Furthermore, our U-Net with ResNet-101 on the D2 dataset achieves the highest accuracy metrics and demonstrates the best inference time among all dataset combinations. This specific result highlights the effectiveness and efficiency of the ResNet-101 backbone within the U-Net architecture when paired with the D2 dataset. It is particularly well-suited for robust and rapid rice disease detection.

### Discussion

#### U-Net with multispectral imagery and vegetation indices for disease detection

In this research, the well-known DL-based semantic segmentation model, U-Net, is employed to detect BLB disease in UAV imagery. Among various combinations, the U-Net architecture trained with the multispectral+NDVI dataset achieves superior segmentation accuracy metrics and provides nearly perfect classification results compared to other combinations, particularly when utilizing ResNet-101 for feature extraction. This indicates that different inputs, specifically various band compositions, can enhance model performance even when using the same architecture [39, 40]. Similarly, a study by Zhang et al. [27] demonstrated that applying U-Net with vegetation indices in the semantic segmentation of wheat yellow rust disease at the field scale in UAV imagery provided high performance and enabled precise classification of disease severity levels. However, the multispectral+NDRE combination does not perform as well as the multispectral+NDVI, highlighting the importance of selecting the appropriate vegetation indices.

#### Superiority of NDVI for BLB detection

Previous literature [22] highlighted NDVI as a robust indicator for detecting rice sheath blight disease compared to other vegetation indices like the Ratio Vegetation Index (RVI), Difference Vegetation Index (DVI), and Normalized Difference Water Index (NDWI). In our study, U-Net architecture incorporating a ResNet-101 backbone with combined multispectral and NDVI imaging proves to be more effective than multispectral+NDRE, demonstrating the effectiveness and simplicity of NDVI for this task. This approach also yields better results than using multispectral alone. Our finding is further supported by experiments with U-Net using a MobileNetV2 backbone and DeepLabV3+ with a ResNet-101 backbone, where both models performed best with the multispectral+NDVI dataset.

#### Challenges in aerial dataset availability

This study highlights the challenge of conducting top-view imaging for rice disease detection using remotely sensed imagery, particularly due to the scarcity of relevant datasets online. Unlike the abundant close-up digital images of rice diseases available on platforms like Kaggle [41], datasets suitable for UAV-based disease detection are much less common [24]. Many related studies, such as [17], resorted to creating their own datasets using disease inoculation techniques. We adapt the same process in our experiment (illustrated in Fig 4). These methods can be not only time-consuming but also limited in coverage to relatively small areas. Such limitations pose significant challenges in achieving the dataset diversity and sufficiency necessary for training effective DL models. Addressing these challenges is critical for advancing the detection of rice disease at the field scale using DL techniques. In the future, the pipeline could be enhanced to diagnose a variety of rice diseases globally, such as Blast, Bacterial Panicle Blight, and Sheath Blight.

#### Addressing data scarcity with augmented pipeline for BLB detection

Our experimental findings introduce a pipeline-based DL technique for detecting rice disease, specifically BLB, in UAV imagery at the field scale. This research provides preliminary guidelines for further studies interested in applying similar methods to other rice diseases. Due to the unavailability of aerial disease datasets, we employ disease inoculation techniques in experimental paddy rice fields. To enhance sample robustness in model training, we apply data augmentation and patch extraction with an overlapping function. Our work addresses knowledge gaps in the application of DL frameworks for BLB detection in rice at the field scale using UAV imagery. This approach offers a viable solution for rice disease prevention and control by mapping field damage due to disease, estimating rice yield losses from BLB, and supporting decision-making processes. It can lead to substantial resource savings, including costs, time, and manpower, thereby enhancing efficiency in agricultural management.

#### Segmentation over object detection in measuring severity and spatial extent for rice BLB detection

While this study focuses on semantic segmentation using U-Net, it is also important to consider the differences and potential advantages of object detection approaches in rice BLB detection. Object detection models, such as YOLO [42] and Faster R-CNN [43], identify and localize disease symptoms by drawing bounding boxes around affected areas. Such techniques can be beneficial in scenarios for detecting and monitoring outbreaks across large areas. However, an object detection is limited to bounding box predictions and cannot measure the precise feature area [44]. At a parcel-level, accurate disease-affected area prediction is crucial for loss assessment and compensation to the farmers. U-Net, a segmentation method, provides pixel-level classification, offering more detailed insights into the spatial extent and severity of the disease.

The choice between segmentation and object detection approaches should depend on the requirements of the agricultural task. For instance, if precise disease mapping and severity assessment are essential, as in this study, segmentation models like U-Net are preferable. However, object detection could be an alternative for broader monitoring for instantaneous identification of diseases over a large area.

## Conclusion

This study presents a robust approach for detecting BLB in rice using a pipeline-based DL technique with UAV imagery. The implementation of the U-Net model with a ResNet-101 backbone, particularly when trained with multispectral and NDVI data, demonstrates superior performance in accurately classifying and assessing the severity of BLB in rice fields. The experiment shows the potential of multispectral+NDVI compared to multispectral-alone and multispectral+NDRE, to enhance model performance even with the same architecture. This method not only improves the precision in identifying disease severity but also aids in segmenting healthy rice from other classes, such as soil, weeds, and water. Therefore, the accurate mapping of the disease extent and severity level in the field is crucial for authorities to accurately determine compensation. Furthermore, the proposed approach offered significant advantages for rice disease prevention and control, and support for decision-making processes. These capabilities can lead to substantial resource savings, including reductions in costs, time, and manpower, thereby enhancing the efficiency of agricultural management.

The scarcity of UAV-based datasets for rice disease detection necessitates the creation of our own dataset using disease inoculation techniques in experimental paddy fields. This approach provides the foundational data required for training and validating our models, despite the challenges in dataset diversity and sufficiency typically faced in this domain. Through data augmentation and patch extraction, we enhance the robustness of our training process, which contributed significantly to the accuracy and reliability of our results. Looking forward, the developed pipeline holds promise for adaptation to other rice diseases, such as Blast, Bacterial Panicle Blight, and Sheath Blight. Future research could focus on extending this methodology to a global scale, incorporating diverse rice disease datasets and further refining the models for broader application in various environmental conditions and agricultural contexts.

## References

[1] U S Department of Agriculture. Rice Sector at a Glance; 2023. https://www.ers.usda.gov/topics/crops/rice/rice-sector-at-a-glance/#Global.

[2] European Commission. INFORM Index for Risk Management; 2023. https://drmkc.jrc.ec.europa.eu/inform-index/INFORM-Risk/Country-Risk-Profile/moduleId/1767/id/386/controller/Admin/action/CountryProfile.

[3] Cao Y, Yuan P, Xu H, Martínez-Ortega JF, Feng J, Zhai Z. Detecting asymptomatic infections of rice bacterial leaf blight using hyperspectral imaging and 3-dimensional convolutional neural network with spectral dilated convolution. Frontiers in Plant Science. 2022;13:963170. doi: 10.3389/fpls.2022.963170 35909723 PMC9328758

[4] LSU AgCenter. Louisiana Rice Production Handbook; 2012.

[5] International Rice Research Institute. Standard Evaluation System for Rice; 2013.

[6] Chandler Jr RF. Rice in the tropics: a guide to development of national programs. CRC Press; 2019.

[7] Ou SH. Rice diseases. IRRI; 1985.

[8] Shahi TB, Xu CY, Neupane A, Guo W. Machine learning methods for precision agriculture with UAV imagery: a review. Electronic Research Archive. 2022;30(12):4277–4317. doi: 10.3934/era.2022218

[9] Li B, Xu X, Han J, Zhang L, Bian C, Jin L, et al. The estimation of crop emergence in potatoes by UAV RGB imagery. Plant Methods. 2019;15:1–13. doi: 10.1186/s13007-019-0399-7 30792752 PMC6371461

[10] Astor T, Dayananda S, Nautiyal S, Wachendorf M. Vegetable crop biomass estimation using hyperspectral and RGB 3D UAV data. Agronomy. 2020;10(10):1600. doi: 10.3390/agronomy10101600

[11] Fu Z, Jiang J, Gao Y, Krienke B, Wang M, Zhong K, et al. Wheat growth monitoring and yield estimation based on multi-rotor unmanned aerial vehicle. Remote Sensing. 2020;12(3):508. doi: 10.3390/rs12030508

[12] Patrick A, Pelham S, Culbreath A, Holbrook CC, De Godoy IJ, Li C. High throughput phenotyping of tomato spot wilt disease in peanuts using unmanned aerial systems and multispectral imaging. IEEE Instrumentation & Measurement Magazine. 2017;20(3):4–12. doi: 10.1109/MIM.2017.7951684

[13] Kerkech M, Hafiane A, Canals R. Deep leaning approach with colorimetric spaces and vegetation indices for vine diseases detection in UAV images. Computers and electronics in agriculture. 2018;155:237–243. doi: 10.1016/j.compag.2018.10.006

[14] Su J, Liu C, Coombes M, Hu X, Wang C, Xu X, et al. Wheat yellow rust monitoring by learning from multispectral UAV aerial imagery. Computers and electronics in agriculture. 2018;155:157–166. doi: 10.1016/j.compag.2018.10.017

[15] Albetis J, Duthoit S, Guttler F, Jacquin A, Goulard M, Poilvé H, et al. Detection of Flavescence dorée grapevine disease using unmanned aerial vehicle (UAV) multispectral imagery. Remote Sensing. 2017;9(4):308. doi: 10.3390/rs9040308

[16] Zhang J, Huang Y, Pu R, Gonzalez-Moreno P, Yuan L, Wu K, et al. Monitoring plant diseases and pests through remote sensing technology: A review. Computers and Electronics in Agriculture. 2019;165:104943. doi: 10.1016/j.compag.2019.104943

[17] Mirandilla JRF, Yamashita M, Yoshimura M, Paringit EC. Leaf Spectral Analysis for Detection and Differentiation of Three Major Rice Diseases in the Philippines. Remote Sensing. 2023;15(12):3058. doi: 10.3390/rs15123058

[18] Lee KD, Park CW, So KH, Na SI. Selection of optimal vegetation indices and regression model for estimation of rice growth using UAV aerial images. Korean Journal of Soil Science and Fertilizer. 2017;50(5):409–421. doi: 10.7745/KJSSF.2017.50.5.409

[19] Mahajan U, Bundel BR. Drones for normalized difference vegetation index (NDVI), to estimate crop health for precision agriculture: A cheaper alternative for spatial satellite sensors. In: Proceedings of the International Conference on Innovative Research in Agriculture, Food Science, Forestry, Horticulture, Aquaculture, Animal Sciences, Biodiversity, Ecological Sciences and Climate Change (AFHABEC-2016), Delhi, India. vol. 22; 2016. p. 31.

[20] Rouse JW, Haas RH, Schell JA, Deering DW, et al. Monitoring vegetation systems in the Great Plains with ERTS. NASA Spec Publ. 1974;351(1):309.

[21] Gitelson AA, Kaufman YJ, Merzlyak MN. Use of a green channel in remote sensing of global vegetation from EOS-MODIS. Remote sensing of Environment. 1996;58(3):289–298. doi: 10.1016/S0034-4257(96)00072-7

[22] Zhang D, Zhou X, Zhang J, Lan Y, Xu C, Liang D. Detection of rice sheath blight using an unmanned aerial system with high-resolution color and multispectral imaging. PloS one. 2018;13(5):e0187470. doi: 10.1371/journal.pone.0187470 29746473 PMC5945033

[23] Ainunnisa I, Haerani H. The identification of pests and diseases of rice plants using sentinel-2 satellite imagery data at the end of the vegetative stage. In: IOP Conference Series: Earth and Environmental Science. vol. 1230. IOP Publishing; 2023. p. 012148.

[24] Zheng Q, Huang W, Xia Q, Dong Y, Ye H, Jiang H, et al. Remote Sensing Monitoring of Rice Diseases and Pests from Different Data Sources: A Review. Agronomy. 2023;13(7):1851. doi: 10.3390/agronomy13071851

[25] Kamilaris A, Prenafeta-Boldu F. Deep learning in agriculture: A survey, computers and electronics in agriculture. 147: 70–90; 2018.

[26] Shamsolmoali P, Zareapoor M, Wang R, Zhou H, Yang J. A novel deep structure U-Net for sea-land segmentation in remote sensing images. IEEE Journal of Selected Topics in Applied Earth Observations and Remote Sensing. 2019;12(9):3219–3232. doi: 10.1109/JSTARS.2019.2925841

[27] Zhang T, Yang Z, Xu Z, Li J. Wheat yellow rust severity detection by efficient DF-UNet and UAV multispectral imagery. IEEE Sensors Journal. 2022;22(9):9057–9068. doi: 10.1109/JSEN.2022.3156097

[28] Oliveira AJ, Assis GA, Faria ER, Souza JR, Vivaldini KC, Guizilini V, et al. Analysis of nematodes in coffee crops at different altitudes using aerial images. In: 2019 27th European Signal Processing Conference (EUSIPCO). IEEE; 2019. p. 1–5.

[29] Su J, Yi D, Su B, Mi Z, Liu C, Hu X, et al. Aerial visual perception in smart farming: Field study of wheat yellow rust monitoring. IEEE transactions on industrial informatics. 2020;17(3):2242–2249. doi: 10.1109/TII.2020.2979237

[30] Ronneberger O, Fischer P, Brox T. U-net: Convolutional networks for biomedical image segmentation. In: Medical image computing and computer-assisted intervention–MICCAI 2015: 18th international conference, Munich, Germany, October 5-9, 2015, proceedings, part III 18. Springer; 2015. p. 234–241.

[31] Chen LC, Zhu Y, Papandreou G, Schroff F, Adam H. Encoder-decoder with atrous separable convolution for semantic image segmentation. In: Proceedings of the European conference on computer vision (ECCV); 2018. p. 801–818.

[32] Zhang Z, Liu Q, Wang Y. Road extraction by deep residual u-net. IEEE Geoscience and Remote Sensing Letters. 2018;15(5):749–753. doi: 10.1109/LGRS.2018.2802944

[33] He K, Zhang X, Ren S, Sun J. Deep residual learning for image recognition. In: Proceedings of the IEEE conference on computer vision and pattern recognition; 2016. p. 770–778.

[34] Zhong Z, Li J, Ma L, Jiang H, Zhao H. Deep residual networks for hyperspectral image classification. In: 2017 IEEE international geoscience and remote sensing symposium (IGARSS). IEEE; 2017. p. 1824–1827.

[35] Shahi TB, Xu CY, Neupane A, Guo W. Recent advances in crop disease detection using UAV and deep learning techniques. Remote Sensing. 2023;15(9):2450. doi: 10.3390/rs15092450

[36] Daglio G, Cesaro P, Todeschini V, Lingua G, Lazzari M, Berta G, et al. Potential field detection of Flavescence dorée and Esca diseases using a ground sensing optical system. Biosystems Engineering. 2022;215:203–214. doi: 10.1016/j.biosystemseng.2022.01.009

[37] Yessou H, Sumbul G, Demir B. A comparative study of deep learning loss functions for multi-label remote sensing image classification. In: IGARSS 2020-2020 IEEE international geoscience and remote sensing symposium. IEEE; 2020. p. 1349–1352.

[38] Sandler M, Howard A, Zhu M, Zhmoginov A, Chen LC. Mobilenetv2: Inverted residuals and linear bottlenecks. In: Proceedings of the IEEE conference on computer vision and pattern recognition; 2018. p. 4510–4520.

[39] Das S, Biswas A, Vimalkumar C, Sinha P. Deep learning analysis of rice blast disease using remote sensing images. IEEE Geoscience and Remote Sensing Letters. 2023;20:1–5. doi: 10.1109/LGRS.2023.3244324

[40] Yang MD, Tseng HH, Hsu YC, Tsai HP. Semantic segmentation using deep learning with vegetation indices for rice lodging identification in multi-date UAV visible images. Remote Sensing. 2020;12(4):633. doi: 10.3390/rs12040633

[41] Rallapalli S, Saleem Durai M. A contemporary approach for disease identification in rice leaf. International Journal of System Assurance Engineering and Management. 2021; p. 1–11.

[42] Redmon J, Divvala S, Girshick R, Farhadi A. You only look once: Unified, real-time object detection. In: Proceedings of the IEEE conference on computer vision and pattern recognition; 2016. p. 779–788.

[43] Ren S, He K, Girshick R, Sun J. Faster R-CNN: Towards real-time object detection with region proposal networks. IEEE transactions on pattern analysis and machine intelligence. 2016;39(6):1137–1149. doi: 10.1109/TPAMI.2016.2577031 27295650

[44] Caldwell DR. Unlocking the mysteries of the bounding box; 2005. ALA Map and Geography Round Table.

